# Expression and Evaluation of Recombinant *Plasmodium knowlesi* Merozoite Surface Protein-3 (MSP-3) for Detection of Human Malaria

**DOI:** 10.1371/journal.pone.0158998

**Published:** 2016-07-08

**Authors:** Jeremy Ryan De Silva, Yee-Ling Lau, Mun-Yik Fong

**Affiliations:** 1 Department of Parasitology, Faculty of Medicine, University of Malaya, 50603 Kuala Lumpur, Malaysia; 2 Tropical Disease Research and Education Centre, Faculty of Medicine, University of Malaya, 50603 Kuala Lumpur, Malaysia; Centro de Pesquisa Rene Rachou/Fundação Oswaldo Cruz (Fiocruz-Minas), BRAZIL

## Abstract

Malaria remains a major health threat in many parts of the globe and causes high mortality and morbidity with 214 million cases of malaria occurring globally in 2015. Recent studies have outlined potential diagnostic markers and vaccine candidates one of which is the merozoite surface protein (MSP)-3. In this study, novel recombinant *Plasmodium knowlesi* MSP-3 was cloned, expressed and purified in an *Escherichia coli* system. Subsequently, the recombinant protein was evaluated for its sensitivity and specificity. The recombinant pkMSP-3 protein reacted with sera from patients with *P*. *knowlesi* infection in both Western blot (61%) and ELISA (100%). Specificity-wise, pkMSP-3 did not react with healthy donor sera in either assay and only reacted with a few non-malarial parasitic patient sera in the ELISA assay (3 of 49). In conclusion, sensitivity and specificity of pkMSP-3 was found to be high in the ELISA and Western Blot assay and thus utilising both assays in tandem would provide the best sero-diagnostic result for *P*. *knowlesi* infection.

## Introduction

Malaria remains a major health threat in many parts of the globe and causes high mortality and morbidity especially in sub-Saharan Africa (90%), according to the World Malaria Report (2015). The same report highlights 214 million cases of malaria occurring globally in 2015 which led to 438000 deaths. The four known species of human malaria parasites are *Plasmodium falciparum*, *Plasmodium vivax*, *Plasmodium malariae*, and *Plasmodium ovale*. *Plasmodium knowlesi* has been recognized as the fifth malaria causing species in humans and has been reported to cause a large number of human infections in South East Asia since the discovery of a large number of infected patients in Borneo, Malaysia in 2004 [1]. Subsequently, increasing numbers of naturally acquired *P*. *knowlesi* infections were found in other Asian countries such as Myanmar, Thailand, Vietnam, the Philippines and Singapore [2–7]. On the other hand, in Peninsular Malaysia more than 300 cases of *P*. *knowlesi* infection have been reported since 2005 [8].

*Plasmodium knowlesi* has a rapid replication cycle and, thus, has been known to cause hyperparasitemia within a short period of time; heightening the severity of the disease. Throughout the course of infection, life-threatening complications such as renal failure, respiratory distress and even death may occur [9]. Compounding the danger of this disease are the difficulties faced when trying to diagnose *P*. *knowlesi* infection. The *P*. *knowlesi* parasite is morphologically similar to both *P*. *falciparum* and *P*. *malariae* under the microscope where early trophozoite of *P*. *knowlesi* parasites resemble that of *P*. *falciparum* and late and mature trophozoites, schizonts, and gametocytes of *P*. *knowlesi* are generally indistinguishable from *P*. *malariae* [10]. Using the polymerase chain reaction (PCR) to detect *P*. *knowlesi* infection presents its own problems as species-specific primers have been reported to cross-react with *P*. *vivax* due to the close phylogenetic relationship between the two species [11].

Recent studies have outlined potential diagnostic markers and vaccine candidates for *P*. *falciparum* infection with clinical trials demonstrated on a variety of antigens; one of which is the merozoite surface protein (MSP)-3 which has partially significant effects on retarding clinical malaria acquisition in children [12–14]. These results confirm the feasibility of an effective malaria vaccine and the prospects that the MSP-3 antigen presents in that regard. MSP-3 was identified in *P*. *falciparum* in 1994 and five years later was identified in *P*. *vivax* [15–17]. Structurally the protein is characterized by a putative signal peptide and lacks a transmembrane domain or a GPI-lipid modification to anchor it to the outer membrane of the parasite. Another characteristic of the protein family is the presence of an alanine-rich central domain containing a series of heptad coiled-coil repeats [15, 18]. Recent studies have predicted that the MSP-3 proteins in *P*. *vivax* form oligometric and elongated molecules that suggest the protein may mediate interactions between host proteins and other merozoite surface proteins [19]. A total of 12 MSP-3 paralogs were identified in the *P*. *vivax* genome indicating this protein belongs to a multi-gene family [20]. An ortholog for the MSP-3 protein has been found in *P*. *knowlesi* and 4 paralogs for this protein have been found in the *P*. *knowlesi* genome showing high similarity to *P*. *vivax* and *Plasmodium cynomolgi* MSP-3 genes [21].

However, in contrast to *P*. *falciparum* and *P*. *vivax* malaria, immunogenicity studies against *P*. *knowlesi* lags far behind. Thus far only a handful of *P*. *knowlesi* recombinant proteins such as the MSP-1_19_, MSP-1_33_ or the surface protein containing an altered thrombospondin repeat domain (SPATR) have been identified and evaluated for potential diagnostic uses or immunogenic properties [22–26]. In this study, novel recombinant *P*. knowlesi MSP-3 protein (pkMSP-3) was cloned, expressed and purified in an *Escherichia coli* system. Subsequently the recombinant protein was evaluated for its sensitivity and specificity using sera from patients infected with knowlesi malaria, non-knowlesi malaria, non-malarial parasitic infections and healthy donor in both an immunoblot and enzyme-linked immunoblot assay (ELISA) to determine its effectiveness as a diagnostic marker.

## Materials and Methods

### Serum collection and ethical consideration

Malaria blood samples were obtained from 68 patients, aged 20 years or older, admitted to the University Malaya Medical Centre (UMMC) in Kuala Lumpur, Malaysia. Blood samples were collected from July 2008 till July 2012. Patients were confirmed for malaria infection by several tests including microscopic examination of Giemsa-stained thick and thin blood smears, BinaxNOW® malaria rapid diagnostic test (Inverness Medical International, Stockport, United Kingdom) and nested PCR based on the *Plasmodium* small subunit ribosomal RNA genes [1]. Ethical approval for this study was obtained from the University of Malaya Medical Centre Ethic Committee (MEC Ref. No. 817.18) and informed verbal consent from the donor or the next of kin was obtained for use of these samples in screening. Written consent was found to be unnecessary as verbal consent would be more sufficient and convenient for the purpose of this study. This is especially so in cases of illiterate patients or where patients only understand a specific language or dialect. In this case, it would be easier to get a translator to convey key information about a project as opposed to having multiple translated copies of written consent forms instead. Patient details were noted down for our personal recordkeeping. This consent procedure was approved by the University of Malaya Medical Centre Ethic Committee. Blood samples from 49 non-malarial parasitic infected patients was also collected and was confirmed by commercial ELISA kits. The patient serum in this study was characterized as follows: *P*. *knowlesi* human malaria; non-*P*. *knowlesi* human malaria (*P*. *falciparum*, *P*. *vivax*, *P*. *ovale*); non-malarial parasitic infection (filariasis, amebiasis, cysticercosis, toxoplasmosis, toxocariasis); and healthy donor serum which was obtained from 56 healthy individuals.

### DNA extraction and construction of recombinant expression plasmid

*Plasmodium knowlesi* DNA was extracted from patient blood sample using a commercial blood extraction kit (Qiagen, Hilden, Germany). A set of primers were designed to allow for PCR product amplification as follows, forward primer MSP3B-F: CCGGATCCATGAAACGCATTTGG and reverse primer MSP3B-R: CGCGGATCCTTACCAGAATTTCAA. Primers were designed based on the *P*. *knowlesi* H strain MSP-3 putative nucleotide sequence, PKH_103010 (Genbank accession no. XM_002259752.1). *BamH*1 restriction sites were introduced into both the forward and reverse sites in order to facilitate cloning. A single step PCR reaction was carried out according to the following protocol: an initial denaturation step at 95°C for 5 min followed by 30 cycles of 94°C for 1 min, 53°C for 1 min and 72°C for 1 min 30 seconds. Finally, an elongation step at 72°C for 10 min was added to the cycle. The PCR product was then purified and cloned into the pGEM-T® TA cloning vector (Promega Corp, USA). The PCR product in the cloning vector was then sent for commercial sequencing to confirm the integrity of the gene by aligning the amplified PCR product nucleotide and amino acid sequences with the deposited putative *pkmsp3* gene, PKH_103010 (XM_002259752.1) in Genbank. Furthermore, the amplified *pkmsp3* sequence was also aligned with MSP-3 orthologues from other *Plasmodium* species to identify percent identity of the nucleotide and amino acid sequences as well as percent of amino acid matches using the Pairwise Sequence Alignment and Clustal Omega tool provided by the European Bioinformatics Institute (EMBL-EBI) Web Services interface (http://www.ebi.ac.uk/Tools/psa/) [27]. Percent of amino acid matches was calculated taking into account fully conserved residues as well as groups of residues with strongly similar properties with a score of greater than 0.5 in the Gonnet PAM 250 matrix [28]. Sequences from *P*. *knowlesi* (Genbank accession no: KT900798-KT900802), *P*. *vivax* (Genbank accession no: KC907580.1-KC907583.1, KC907572.1), *P*. *cynomolgi* (Genbank accession no: KC907501.1-KC907504.1, KC907501.6) and *P*. *falciparum* (Genbank accession no: AM161584.1, AM161586.1, AM161615.1, AM161618.1, AM161642.1) were retrieved from Genbank and used to construct a phylogenic tree using the neighbour-joining method in MEGA6 computational program [29]. Bootstrap proportions were used to assess the robustness of the tree with 1000 bootstrap replicates. The putative size of *P*. *knowlesi* MSP-3 was calculated based on the *pkmsp3* nucleotide sequence deposited in Genbank utilizing Generunner software version 4.0.9.2 (Hastings Software Inc., Hastings, NY, USA; http://www.generunner.net/). After confirmation of the sequences the *pkmsp3* recombinant plasmid was then digested with the *BamH*1 restriction enzyme and cloned into the T7 promoter-based pRSET A vector; which allows for expression of dually polyhistidine (His)- and Xpress epitope-tagged recombinant proteins in *E*. *coli* (Invitrogen, USA). Successfully cloned recombinant pkMSP-3 expression vector was then again sent for sequencing to confirm the presence, integrity, and orientation of the *pkmsp3* gene before expression. Transformation into maintenance host *E*. *coli* TOP10F’ strain was performed for propagation and maintenance of the plasmid. Before expression, the plasmid was transformed into expression host *E*. *coli* BL21 (DE3) pLysS strain.

### Expression of recombinant pkMSP-3

A single recombinant BL21 (DE3) pLysS clone was inoculated and propagated overnight in Luria Bertani (LB) broth containing ampicillin (100 μg/mL) and chloramphenicol (35 μg/mL) at 37°C with shaking. The overnight culture was then further diluted with LB broth to an optical density (OD) at 600 nm of 0.1 and the culture was then allowed to grow to an OD of 0.4 to 0.6. This was followed by induction of overexpression of the pkMSP-3 protein by the addition of 1 mM isopropyl-1-thio-D-galactopyranoside (IPTG) for 2 hours. Cells were then pelleted at 5000 rpm for 10 min. The supernatant collected was discarded and the cell pellet was then re-suspended in 5 mL of Bug Buster reagent (Novagen, USA) per gram of wet cell pellet. One μl of Benzonase (50 mM Tris-HCL, pH 8.0, 20 mM NaCL, and 2 mM MgCl_2_ in 50% glycerol) was added per mL of Bug Buster reagent used. The solution containing the cell pellet was allowed to incubate on a rocker till the cell pellet was fully lysed. Insoluble cell debris was separated through centrifugation at 16000 x g for 15 min at 4°C. The insoluble cell pellet was then further processed using 200 μg/mL lyzosyme in the same volume of Bug Buster reagent that had been used during the initial resuspension of the cell pellet. The suspension in lysozyme was then allowed to incubate at room temperature for 10 min and was then further centrifuged at 16000 x g for 15 min at 4°C. The pellet was then washed with 1:10 diluted Bug Buster reagent and the washing was repeated 3 times. The final inclusion body pellet was then re-suspended in Phosphate Buffered Saline (PBS), and a fraction was used for sodium dodecyl sulphate polyacrylamide gel electrophoresis (SDS-PAGE) and Western blotting.

### Optimization of recombinant pkMSP-3 expression conditions

Optimum expression conditions for maximizing the protein yield was studied by monitoring expression levels of a number of variables including concentration of IPTG used for induction and collection time after induction. The IPTG concentrations studied were 0.5 mM, 0.75 mM, 1.0 mM, and 2.0 mM (S1 and S3 Figs). Cell culture of 1 mL in volume was collected before induction and every hour after induction up to 4 hours. Aside from this, expression levels with induction at different OD was also studied in an effort to identify optimal expression conditions (S2 and S4 Figs). Cells were harvested and analysed by SDS-PAGE and Western blotting.

### SDS-PAGE, Coomassie brilliant blue staining and Western blot

Recombinant pkMSP-3 were resolved by 12% SDS-PAGE and stained with Coomassie brilliant blue (Bio-Rad, USA). The separated proteins were also electrophorectically transferred onto polyvinylidene difluoride (PVDF) membranes (Bio-Rad, USA) with protein amounts ranging between 50 to 300 ng and blocked overnight in Tris Buffered Saline (TBS) containing 5% skimmed milk at 4°C. The membranes were washed three times with TBS containing 0.2% Tween-20 (TBS-T) and probed with either anti-Xpress™ antibody (1:5000 dilution) (Invitrogen, USA) or with patient serum (1:200 dilution) with TBS containing 2.5% skimmed milk for two hours with constant shaking at room temperature. After three washes with TBS-T the membrane was treated with biotin-labelled goat anti-mouse IgG (1:2500 dilution) if using anti-Xpress™ antibody; or biotin labelled goat anti-human IgA plus IgM plus IgG (1:2500 dilution) is using human serum (Kierkegaard and Perry Inc., USA) for one hour, followed by streptavidin-alkaline phosphatase (1: 2500 dilution) for one hour. Finally, the membranes were developed by chromogenic nitro-blue tetrazolium/5-bromo-4-chloro-3’-indolyphosphate substrate. The colour was allowed to develop at room temperature in the dark.

### Purification of recombinant pkMSP-3

Cell pellets from 50 ml of recombinant pkMSP-3 protein cultures were re-suspended in 8 mL of 6 M urea and added to a ProBond column (Invitrogen, USA) filled with 2 mL of Nickel-NTA resin. The column was then loaded into a MACSmix™ Tube Rotator (Miltenyi Biotec, Germany), and the His-tagged protein was then allowed to bind to the Ni-NTA resin for 4 hours at 4°C. The column is then moved onto a stand and the resin was allowed to settle to the bottom of the column. The supernatant was then aspirated gently and the column was washed with 8 mL of 6 M urea. Washing was done by inverting the tube 10 times to allow the resin to mix with the urea and the column was then returned to the stand to allow the resin to settle. The supernatant is once again aspirated and replaced with 4 M urea. Washing was then repeated and this step was further carried out using 2 M urea. Finally, the protein was eluted with 5 mL of 1 M urea and 250 mM imidazole. The purity of recombinant pkMSP-3 was determined via SDS-PAGE.

### Refolding of pkMSP-3

Refolding of the pkMSP-3 protein was carried out via dialysis in PBS as described by Chao *et al* [30]. The purified polypeptides at a concentration of approximately 0.8 mg/mL were transferred into dialysis cassettes with a molecular weight cut-off point of 10 kDa and dialyzed against PBS for up to 16 hours with 2–4 buffer changes during the duration of dialysis. Usually one dialysis cassette, with 20 mL each in a 1,000-mL beaker was dialyzed against buffer at a ratio of 1:35 of sample volume to dialysis buffer. All dialysis procedures were conducted at 4°C. The dialysis was left overnight in large excess of PBS at 4°C to remove traces of urea. The dialyzed protein was then quantified using a Bradford Assay Kit with Bovine Serum Albumin (BSA) as standard (Bio-Rad, USA) before subsequent downstream experiments. The pkMSP-3 dialyzed protein was evaluated for native refolding and native confirmation via a reducing assay using β-mercaptoethanol as a reducing agent. Recombinant pkMSP-3 protein was boiled in reducing and non-reducing sample buffer with and without β-mercaptoethanol respectively. Samples were then run on an SDS-PAGE gel, stained with Coomassie blue, and native confirmation was evaluated via a difference in mobility in the gel (S5 Fig).

### Evaluation of recombinant pkMSP-3 in Western Blot assay

The purified recombinant pkMSP-3 was tested by Western Blotting using serum samples from patients infected with *P*. *knowlesi* (n = 41) and non-*P*. *knowlesi* human *Plasmodium* species, which include *P*. *falciparum* (n = 11), *P*. *vivax* (n = 15), and *P*. *ovale* (n = 1). In addition, serum samples from patients with non-malarial parasitic infections which include amebiasis (n = 15), toxoplasmosis (n = 16), filariasis (n = 4), cysticercosis (n = 12), toxocariasis (n = 2), and healthy donor (n = 56) were also included in the assays.

### Evaluation of recombinant pkMSP-3 in ELISA

A 96-well microtiter plate (TPP, Trasadingen, Switzerland) was coated with 10 μg/mL of pkMSP-3 recombinant protein diluted using 0.05 M sodium carbonate buffer, pH 9.6 and incubated overnight at 4°C. Each well was incubated with 50 μl of diluted pkMSP-3 for a final concentration of 500 ng of pkMSP-3 protein per well. The wells were then washed with PBS containing 0.1% Tween-20 a total of three times. Blocking buffer (1% BSA in PBS) was added to each well and the plate was left to incubate for 2 hours at 37°C. The wells were further washed 3 times and then incubated with patient serum in a 1:80 dilution of 1% BSA/PBS for an hour at 37°C. Consequently, the wells were then washed 5 times to remove any patient serum left in the well and 1:2500 diluted secondary antibody consisting of peroxidase-labelled goat anti-human IgG (Biorad, USA) was added to each well and the plate was incubated for 1 hour at 37°C. The wells were washed 5 times for a final time after incubation and incubated with 3, 3’, 5, 5’–tetramethyl benzidine (TMB) (Amresco, Solon, OH) for 30 min in the dark to allow colour to develop. The reaction was stopped with 2 N H_2_SO_4_. The OD was read at 450 nm and determined by subtracting the OD of the blank containing TMB from the mean OD of the sample. All samples were run in triplicates and the mean OD of each sample was used for further analysis. The cut-off value was calculated as the M_N_ + 2σ of the healthy donor serum group where M_N_ and σ are the mean OD and standard deviation respectively. Samples with a mean OD greater than the cut-off point were considered positive and vice-versa.

### Statistical analysis

The sensitivity and specificity of both Western blotting and ELISA for the detection of malarial infection was calculated by using the following formulae: TP/(TP+FN) x 100% for sensitivity and TN/(TN+FP) x 100% for specificity where TP = true positive, TN = true negative, FP = false positive, FN = false negative for malarial infection. The gold standard used for comparison in the analysis of sensitivity and specificity was microscopic analysis of thick and thin smears as well as PCR.

## Results

### Cloning and expression of recombinant pkMSP-3

**Fig 1:**
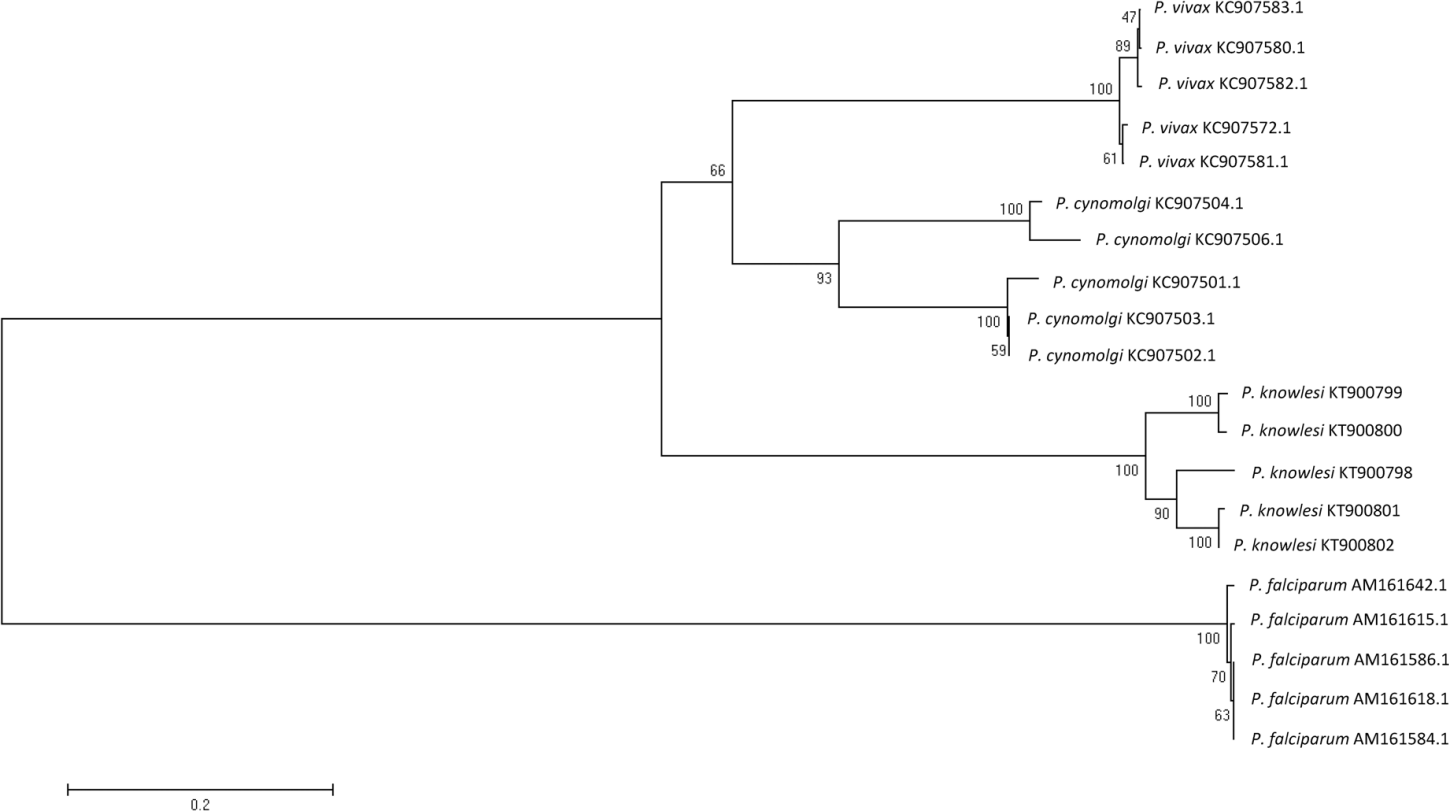
Phylogenetic tree of related *Plasmodium spp*. inferred from *msp3* sequences The tree was constructed using a neighbour-joining method with the MEGA6 program. The number on the branches indicate the bootstrap proportions (1000 replicates)

The *P*. *knowlesi* MSP-3 protein was successfully amplified from genomic DNA by PCR and had an expected size of approximately 1077 base pairs. The gene was cloned into the pRSET A expression vector and transformed into BL21 (DE3) pLysS host cell. Recombinant plasmids were isolated and sequenced to confirm the identity and integrity of the cloned gene before expression. The nucleotide and amino acid sequence percent identity of the *pkmsp3* gene was calculated relative to the *msp3* of closely related *Plasmodium spp*. as seen in Table 1. *Plasmodium knowlesi msp3* had nucleotide and amino identities of >50% with both *P*. *vivax* and *P*. *cynomolgi msp3*. A phylogenetic tree seen in Fig 1 was also constructed using sequenced *pkmsp3* genes and *P*. *vivax*, *P*. *cynomolgi* and *P*. *falciparum msp3* genes retrieved from Genbank. It was observed that the sequences clustered into respective *Plasmodium* groups with *pkmsp3* genes branching out closer to the *P*. *vivax* and *P*. *cynomolgi msp3* groups as compared to *P*. *falciparum* which branches out further away. The high bootstrap value validates the branching that is observed in the phylogenetic tree.

**Table 1:** *Plasmodium knowlesi* MSP-3 nucleotide and amino acid identities and amino acid matches compared with other related *Plasmodium* species*

*P*. *knowlesi*	Nucleotide identities (%)	Amino acid identities (%)	Amino acid matches (%)
*P*. *vivax*	57.4%	37.4%	51.8%
*P*. *falciparum*	43.6%	12.0%	22.8%
*P*. *cynomolgi*	70.4%	50.5%	65.5%

* The percent identity and amino acid matches was determined by pairwise sequence alignment of *pkmsp3* sequence with orthologous *msp3* from other *Plasmodium* species. Amino acid matches was calculated based on conserved amino acid residues or groups of residues with strongly similar properties.

**Fig 2:**
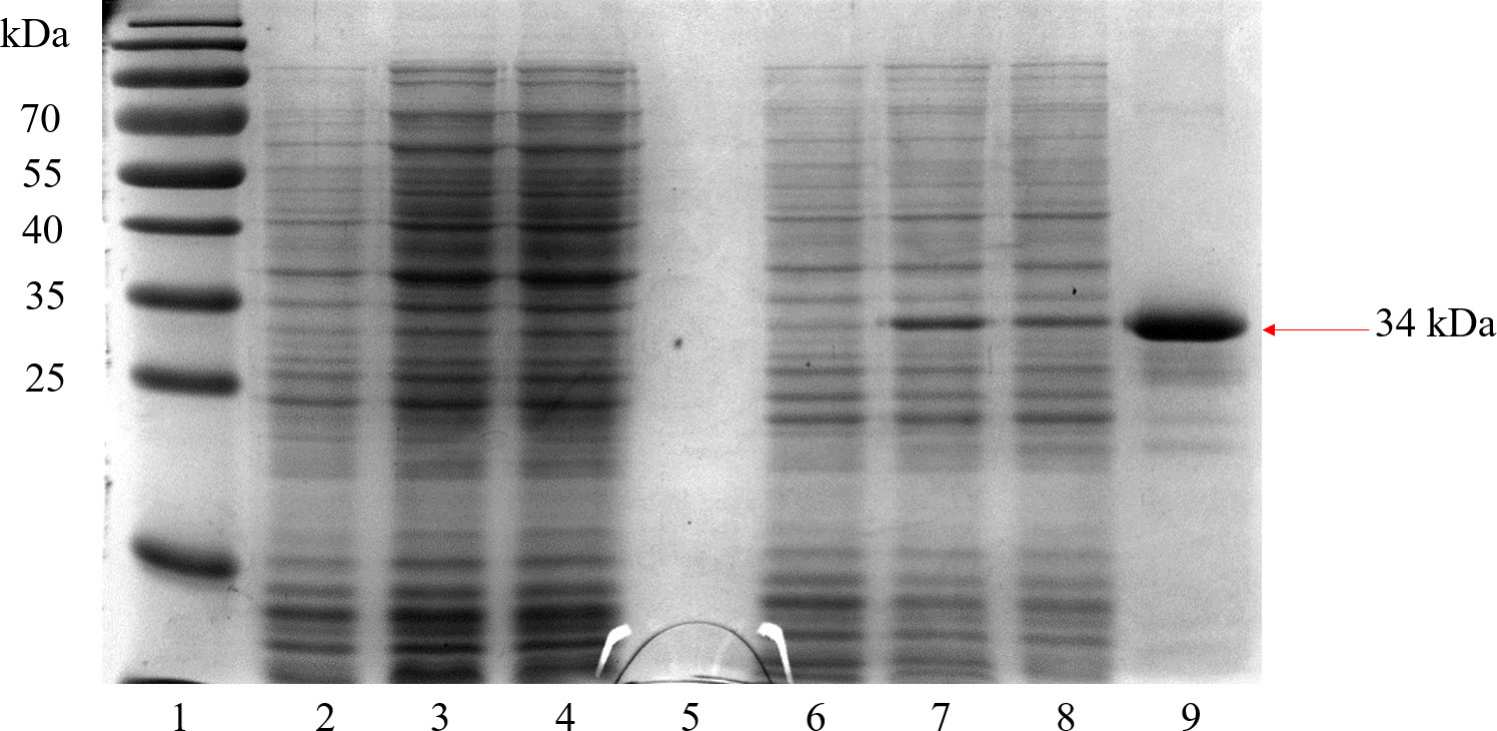
Sodium dodecylsulfate-polyacrylamide gel electrophoresis analysis of expression and Ni-NTA purified recombinant *Plasmodium knowlesi* Merozoite Surface Protein-3 (pkMSP-3) protein in *Escherichia coli* BL21 (DE3) pLysS with an empty vector control at different hours of expression. Lane 1, Bio-Rad (Hercules, CA) Pre-stained Broad Range Protein Ladder; Lane 2–4, negative control pRSET A clone at 0,2, and 4 hours after induction with 1 mM isopropyl β-D-1-thiogalactopyranoside (IPTG); Lane 5, purification of a 50 mL culture of negative control pRSET A with nickel resin using the urea denaturing method of purification; Lane 6–8, pkMSP-3 clone at 0, 2, and 4 hours after induction with 1 mM IPTG; Lane 9, purification of a 50 mL culture of recombinant pkMSP-3 protein. The arrow indicates expression of pkMSP-3 at expected size (34 kDa). A 50 mL culture of recombinant pkMSP-3 yielded ~0.2 mg/mL of protein.

The recombinant pkMSP-3 clone was able to successfully exhibit a high level of expression of the pkMSP-3 protein with an approximate size of 34 kDa indicated by the thick band visible in Fig 2 which is not seen in the non-recombinant plasmid control.

Determination of optimal expression conditions was carried out before large scale expression and purification to maximize the yield of protein. A time-course experiment was carried out to determine the optimum length of expression. In tandem with that, different concentrations of IPTG were used (0.5 mM, 0.75 mM, 1.0 mM and 2.0 mM). We found that differing concentrations of IPTG did not yield a significant difference in protein as observed on a Coomassie-stained gel at the same time point. We also observed no difference in protein yield between expression times of 2 hours and 4 hours. Thus, we concluded that the best expression conditions for maximum yield of recombinant pkMSP-3 would be for 2 hours at 37°C with 1.0 mM IPTG used for induction and constant shaking at 250 rpm.

### Purification of recombinant pkMSP-3

**Fig 3:**
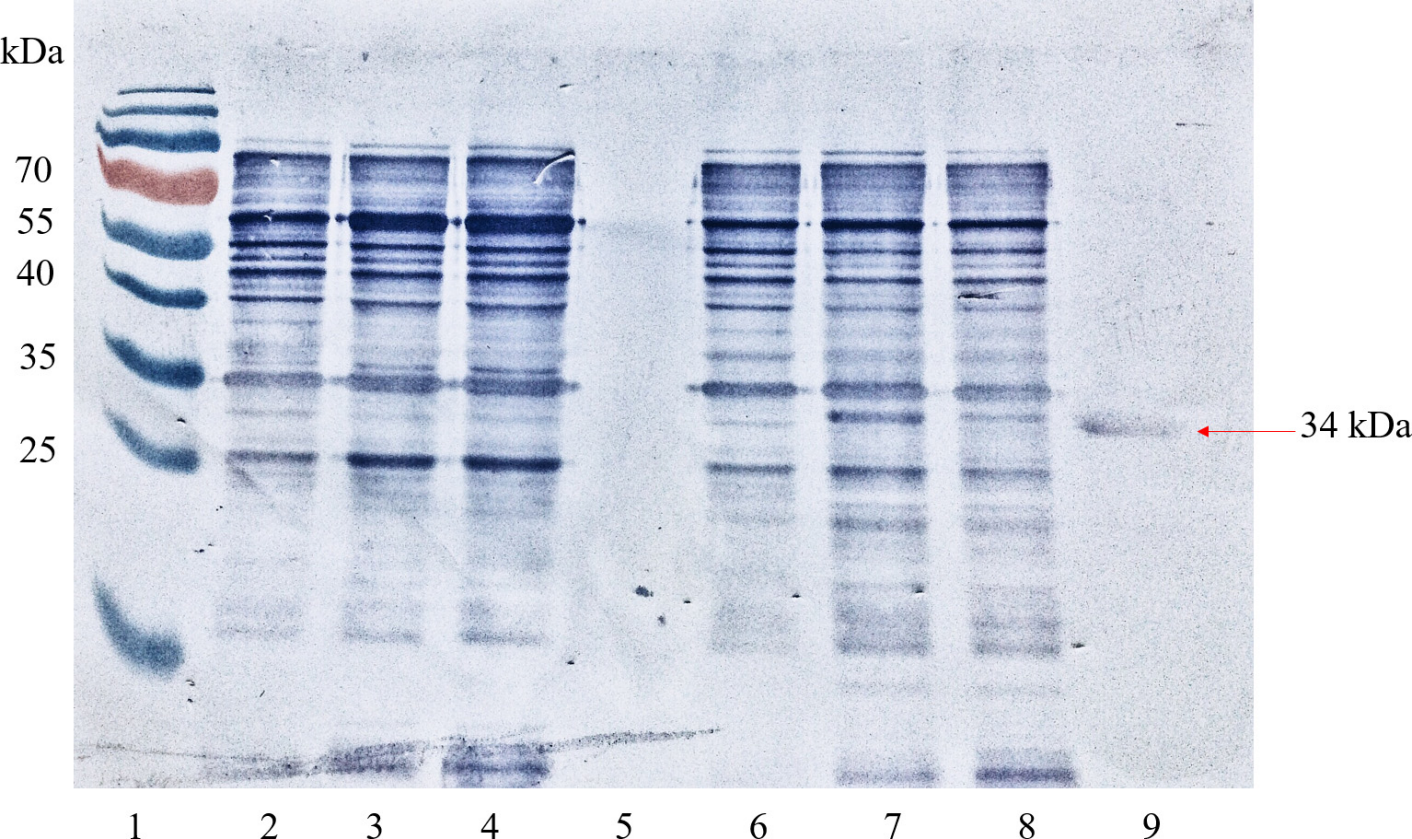
Western blot assay analysis of expression and Ni-NTA purified recombinant *Plasmodium knowlesi* Merozoite Surface Protein-3 (pkMSP-3) protein in *Escherichia coli* BL21 (DE3) pLysS with an empty vector control at different hours of expression. Lane 1, Bio-Rad (Hercules, CA) Pre-stained Broad Range Protein Ladder; Lane 2–4, negative control pRSET A clone at 0,2, and 4 hours after induction with 1.0 mM isopropyl β-D-1-thiogalactopyranoside (IPTG); Lane 5, purified negative control, pRSET A; Lane 6–8, pkMSP-3 clone at 0,2, and 4 hours after induction with 1.0 mM IPTG; Lane 9, purified recombinant pkMSP-3 protein. The arrow indicates expression of pkMSP-3 at expected size (34 kDa). A 50 mL culture of recombinant pkMSP-3 yielded 10 mg of protein.

Recombinant pkMSP-3 was successfully purified under denaturing conditions using a Ni-NTA column. Purified products were observed as a single band at 34 kDa in both a Coomassie-stained SDS-PAGE gel (Fig 2) and a Western blot using anti-Xpress as the primary antibody (Fig 3). Subsequent, quantification of pkMSP-3 protein indicated a concentration of protein that ranged from 0.7–0.8 mg/mL for a 400 mL culture.

### Western blot analysis of recombinant pkMSP-3 with human serum samples

**Fig 4:**
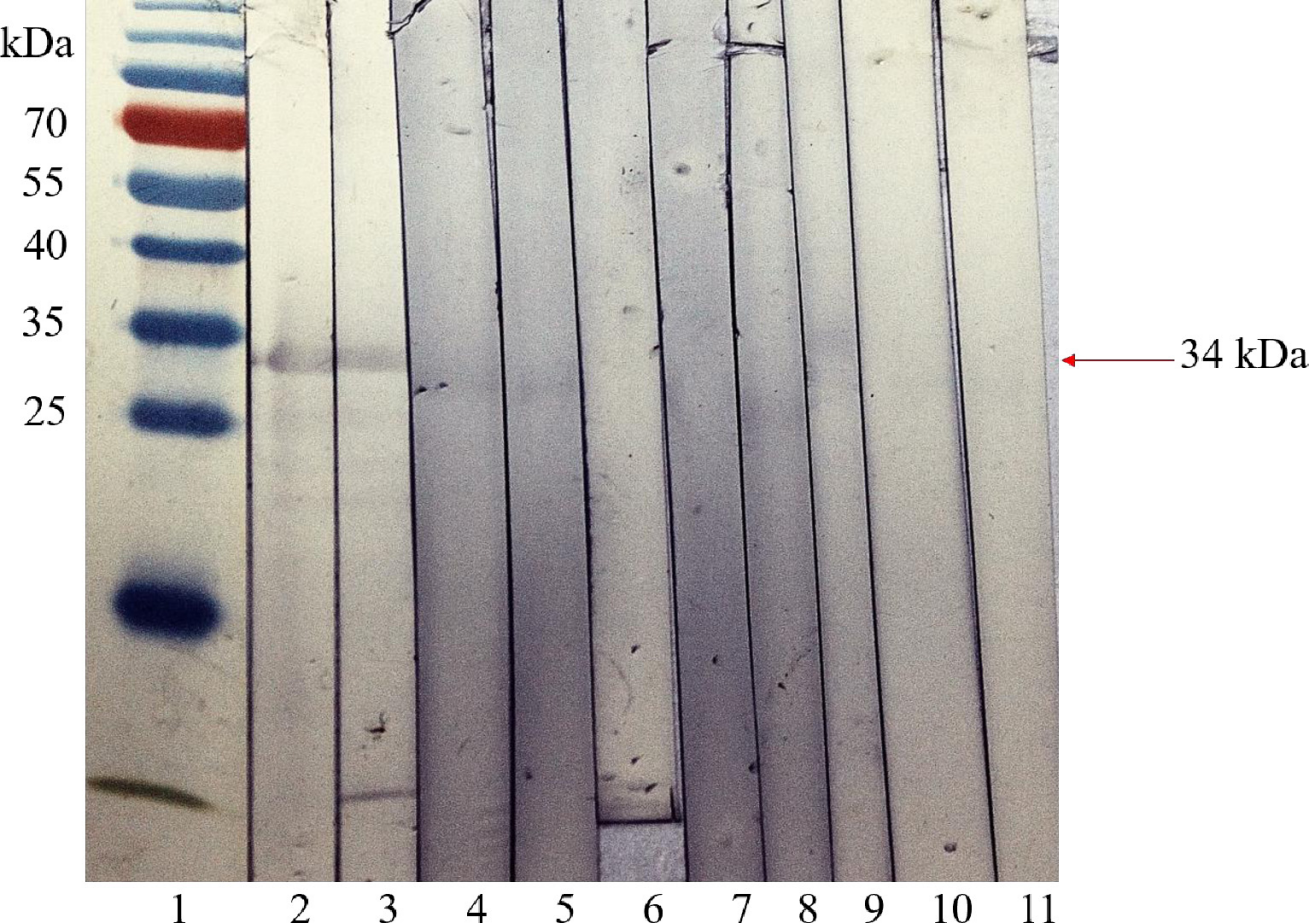
Detection of recombinant *Plasmodium knowlesi* merozoite surface protein-3 (pkMSP-3) with patient serum samples infected with *P*. *knowlesi* and other parasite species. Each western strip contained roughly 70 ng of purified pkMSP-3 and was tested with serum from each category. Selected samples from each category are shown. Lane 1, Bio-rad (Hercules, CA) Pre-stained Broad Range Protein Ladder; Lane 2–3, serum of patients infected with *P*. *knowlesi*; Lane 4, serum of patients infected with *P*. *falciparum*; Lane 5, serum of patients infected with *P*. *vivax*. Lane 6–10 contained sera of patients infected with non-malarial parasites. Lane 6, filariasis; Lane 7, amebiasis; Lane 8, toxoplasmosis; Lane 9, cysticercosis; Lane 10, toxocariasis. Lane 11 contained healthy donor serum which served as a negative control.

The purified pkMSP-3 was evaluated by Western Blotting using serum samples from patients with *P*. *knowlesi* human malaria, non-*P*. *knowlesi* human malaria, non-malarial parasitic infections and healthy donors (Fig 4). The recombinant pkMSP-3 reacted with 61.0% of *knowlesi* malaria serum samples (25/41) and 14.8% of non-*knowlesi* malaria serum samples which comprised of 4 *P*. *vivax* patient sera. None of the non-malaria or healthy donor serum samples picked up pkMSP-3 thus giving the protein a specificity of 100% (105/105).

### ELISA analysis of recombinant pkMSP-3 with human serum samples

The same serum samples used in Western blot were also used in ELISA. For ELISA, 10 μg/mL of recombinant pkMSP-3 was coated in each well and a 1:80 dilution of patient sera was used per patient. The recombinant pkMSP-3 protein reacted with all 41 *knowlesi* patient serum giving it 100% sensitivity to *P*. *knowlesi* patient sera. It also reacted with 70.4% or 19 of 27 non-*knowlesi* malaria samples; predominantly with *P*. *vivax* samples where 11 of the 15 *P*. *vivax* samples picked up pkMSP-3. Three non-malarial parasitic patient sera were found to interact with pkMSP-3 indicating a specificity of 97.1%. Results of the patient sera screening against recombinant pkMSP-3 in Western blot and ELISA are shown in Table 2. Sensitivity and specificity of the Western blot assay and ELISA assay are shown in Table 3.

**Table 2:** Detection of recombinant pkMSP-3 with patient serum infected with *Plasmodium knowlesi* and other parasite species in Western Blot and ELISA*

Human Sera Group	Number of Sera Tested	Western blot		ELISA	
		Positive	Negative	Positive	Negative
		No.	No.	No.	No.
A. *Plasmodium knowlesi*	41	25	16	41	0
B. Non-*P*. *knowlesi* human malaria					
i. *P*. *vivax*	15	4	11	11	4
ii. *P*. *falciparum*	11	0	11	8	3
iii. *P*. *ovale*	1	0	1	0	1
C. Non-malarial parasitic infection					
i. Filariasis	4	0	4	0	4
ii. Amebiasis	15	0	15	1	14
iii. Cysticercosis	12	0	12	1	11
iv. Toxoplasmosis	16	0	16	1	15
v. Toxocariasis	2	0	2	0	2
D. Healthy donor	56	0	56	0	56

*pkMSP-3 = recombinant *Plasmodium knowlesi* merozoite surface protein-3, ELISA = enzyme-linked immunosorbent assay.

**Table 3:** Sensitivity and specificity of Western blot assay and ELISA using recombinant pkMSP-3*.

	Sensitivity (%)		
Assay	*Plasmodium knowlesi infection*	Non-*P*. *knowlesi malarial infection*	Specificity (%)
Western Blot	61.0	14.8	100.0
ELISA	100.0	70.4	97.1

*pkMSP-3 = recombinant *Plasmodium knowlesi* merozoite surface protein-3, ELISA = enzyme-linked immunosorbent assay.

## Discussion

The *P*. *knowlesi* parasite is able to replicate and complete its blood stage life cycle within 24 hours, the shortest of all human *Plasmodium*, and thus it is necessary to devise a diagnostic tool that is both sensitive and specific to *P*. *knowlesi*. Thus, we sought out to evaluate the sero-diagnostic potential of *P*. *knowlesi* MSP-3; a previously unstudied protein to assess its efficacy.

For the purposes of this study we opted to use the *E*. *coli* system for production of the recombinant pkMSP-3. This was due to the ease of use, safety, and known genetic properties of *E*. *coli* that make it such an appealing expression host. Furthermore, *E*. *coli* is well established and is receptive to foreign DNA when manipulated by genetic manipulation methods. It has the advantage of being able to produce large amounts of protein in an efficient and inexpensive manner which increases its appeal in lieu of other alternatives such as yeast or mammalian cell expression.

The use of recombinant protein can be justified by how advantageous it is in terms of how rapid and simple recombinant protein can be expressed and obtained. This is in light of the fact that the difficulties in long-term culture of *P*. *knowlesi* coupled with the complexities of extracting and preparing parasite lysate protein make recombinant *P*. *knowlesi* protein an ideal alternative for sustainable production of large amounts of pkMSP-3. The recombinant pkMSP-3 protein was recovered as inclusion bodies expressed in *E*. *coli* and there are several reasons that may have contributed to this inclusion body formation. It has been shown that expression of genes that have a large evolutionary distance between the native host and expression host may have a higher propensity for inclusion body formation [31]. This may also extend here where a protein found natively in *P*. *knowlesi* is expressed in a non–native *E*. *coli* host instead. Furthermore, the protein structure of pkMSP-3 with its alanine-rich central domain increases the overall hydrophobicity of the protein which greatly increases the propensity for inclusion body formation [32]. The inclusion bodies purified in this study were denatured using the Bug Buster protein extraction kit, and reduction in urea concentration though dialysis against PBS was used to refold the denatured protein [30]. Refolded protein was evaluated using a β-mercaptoethanol reducing assay. In our study, no difference of mobility in the gel was observed between samples boiled in reducing or non-reducing buffer. This was also observed in the native non-solubilized protein. This may be attributed to the fact that pkMSP-3 does not possess any cysteine amino acid residues. Thus, native confirmation of the refolded pkMSP-3 protein through a reducing assay would not be suitable. An alternative to this would be a functional assay to prove that protein structure and function has been restored. Our preliminary observations indicate that mice injected with the pkMSP-3 refolded protein yielded antibodies that were able to recognize antigens on the surface of native *P*. *knowlesi* parasites in an immunofluorescence (IFA) assay (Unpublished data). Ideally, crystallization or mass-spectrometry studies for protein refolding are the best option, however, this is one of the limitations of this study due to equipment and facility constraints.

During screening of various patient sera using pkMSP-3 recombinant protein we found high sensitivity of the protein (>90%) in the ELISA assay but relatively lower sensitivity in Western blot. This may be due to the loss of protein antigenicity during the denaturing electrophoretic preparation of the antigen for Western blot which does not occur in the ELISA assay allowing preservation of conformational epitopes [33, 34]. Furthermore, high-molecular-weight proteins transfer poorly to nitrocellulose filters in immunoblot, preventing recognition by the antibodies [35]. In our studies it was also observed that some *P*. *knowlesi* patient sera that was used for ELISA screening had borderline activity, where the OD absorbance value of the patient sera hovered slightly above the cut-off value and this may have led to discrepancies in the result [24, 36]. Another explanation for the non-reactivity of certain malaria-infected patient sera with pkMSP-3 in both assays is that in certain cases the sera was collected from the patient before adequate production of antibodies against the invading malaria parasite has commenced [37]. Specificity of the pkMSP-3 however, was relatively high (>90%) for both assays indicating that this may be a promising and reliable antigen for use in sero-detection of malarial infection.

It was found that particularly in the ELISA assay, pkMSP-3 cross-reacted with other non-*knowlesi* malaria sera and this could be due to serological cross-reactivity where previous studies have described cross-reactivity could occur among the *Plasmodia* family [38–40]. In addition to that, it was observed that the *pkmsp3* gene possessed high nucleotide and amino acid identities to other members of the *Plasmodium* species including *P*. *vivax* (57.4%) and *P*. *cynomolgi* (70.4%) (Table 1). This is also observed in the phylogenetic tree constructed using *pkmsp3* gene sequences where *pkmsp3* sequences were seen to branch out closer to *P*. *vivax msp3* and *P*. *cynomolgi msp3* sequences (Fig 1) and thus tallies with the nucleotide and amino acid identity data in Table 1. Antibodies generated against a specific protein may often interact with other closely related proteins with equal amino acid identities and this has been observed in other *P*. *knowlesi* protein evaluation studies [22, 24]. Cross-reactivity in both assays to non-malarial and non-knowlesi malaria patient sera may also be due to previous exposure of the patients to *P*. *knowlesi* infection which is a possibility we have not ruled out. Indeed, it is not unheard of, as a study done by Wipasa *et al* has demonstrated that antibodies generated against the malaria parasite during infection are able to last in the human body for a period of 5 years or more [41].

It is noteworthy that, as cross-reactivity was mostly seen in the ELISA assay and not in Western blot it also may be attributed to borderline activity of the sera resulting in a false positive result. There are however, several advantages to utilising the ELISA assay over Western blot assays as an optimized ELISA assay uses marginally smaller amounts of serum for each reaction which is useful when dealing with small volume samples. Throughput is also usually higher and results can be obtained faster by ELISA as compared to Western Blot and this is critical in diagnosis. Lastly, ELISA assays have the ability to quantitatively determine the antibody titer of the serum by measuring the OD value of the sera which correlates to antibody titer.

## Conclusion

In conclusion, the recombinant pkMSP-3 protein was successfully expressed and purified in *E*. *coli* under optimal conditions. Sensitivity was found to be high when evaluated with an ELISA assay and specificity was found to be high when evaluated using Western Blot indicating that utilising both assays in tandem would provide the best sero-diagnostic results for detecting human malarial infection. Cross-reactivity was observed in evaluation of this recombinant protein and as such further evaluation with non-knowlesi MSP-3 proteins would be ideal. As a future study, it would be interesting to compare and evaluate the results obtained in this study with the *P*. *vivax* MSP-3 protein in terms of sensitivity and specificity. Further studies including protein crystallization, mass spectrometry and immunocharacterization of this protein should be carried out to assess its potential as a vaccine candidate.

## Supporting Information

S1 Fig Sodium dodecylsulfate-polyacrylamide gel electrophoresis analysis of expression and Ni-NTA purified recombinant *Plasmodium knowlesi* Merozoite Surface Protein-3 (pkMSP-3) protein in *Escherichia coli* BL21 (DE3) pLysS with an empty vector control at different concentrations of isopropyl β-D-1-thiogalactopyranoside (IPTG). Lane 1, Bio-Rad (Hercules, CA) Pre-stained Broad Range Protein Ladder; Lane 2–5, negative control pRSET A clone induced with 0.5 mM, 0.75 mM, 1 mM and 2.0 mM IPTG; Lane 6–9, pkMSP-3 clone induced with 0.5 mM, 0.75 mM, 1 mM and 1.5 mM IPTG; The arrow indicates expression of pkMSP-3 at expected size (34 kDa) with optimum expression at 1 mM IPTG with no obvious increase in expression at higher concentrations.
(TIF)

S2 Fig Sodium dodecylsulfate-polyacrylamide gel electrophoresis analysis of expression and Ni-NTA purified recombinant *Plasmodium knowlesi* Merozoite Surface Protein-3 (pkMSP-3) protein in *Escherichia coli* BL21 (DE3) pLysS with an empty vector control with induction at different optical density (OD). Lane 1, Bio-Rad (Hercules, CA) Pre-stained Broad Range Protein Ladder; Lane 2–5, negative control pRSET A clone induced at OD 0.4, OD 0.6, OD 0.8 and OD 1.0 with 1 mM isopropyl β-D-1-thiogalactopyranoside (IPTG); Lane 6–8, pkMSP-3 clone induced at OD 1.0, OD 0.6, and OD 0.4 with 1 mM (IPTG); The arrow indicates expression of pkMSP-3 at expected size (34 kDa) with optimum expression at 1 mM IPTG.
(TIF)

S3 Fig Western blot analysis of expression and Ni-NTA purified recombinant *Plasmodium knowlesi* Merozoite Surface Protein-3 (pkMSP-3) protein in *Escherichia coli* BL21 (DE3) pLysS with an empty vector control at different concentrations of isopropyl β-D-1-thiogalactopyranoside (IPTG). Lane 1, Bio-Rad (Hercules, CA) Pre-stained Broad Range Protein Ladder; Lane 2–5, negative control pRSET A clone induced with 0.5 mM, 0.75 mM, 1 mM and 2.0 mM IPTG; Lane 6–9, pkMSP-3 clone induced with 0.5 mM, 0.75 mM, 1 mM and 1.5 mM IPTG; The arrow indicates expression of pkMSP-3 at expected size (34 kDa).
(TIF)

S4 Fig Western blot analysis of expression and Ni-NTA purified recombinant *Plasmodium knowlesi* Merozoite Surface Protein-3 (pkMSP-3) protein in *Escherichia coli* BL21 (DE3) pLysS with an empty vector control with induction at different optical density (OD). Lane 1, Bio-Rad (Hercules, CA) Pre-stained Broad Range Protein Ladder; Lane 2–5, negative control pRSET A clone induced at OD 0.4, OD 0.6, OD 0.8 and OD 1.0 with 1 mM isopropyl β-D-1-thiogalactopyranoside (IPTG); Lane 6–8, pkMSP-3 clone induced at OD 1.0, OD 0.6, and OD 0.4 with 1 mM (IPTG); The arrow indicates expression of pkMSP-3 at expected size (34 kDa).
(TIF)

S5 Fig SDS-PAGE gel of pkMSP-3 in reducing and non-reducing sample buffer. Lane 1 contains native pkMSP-3 before solubilisation and refolding in non-reducing sample buffer. Lane 2 contains native pkMSP-3 before solubilisation and refolding in standard reducing sample buffer. Lane 3 contains refolded pkMSP-3 in non-reducing sample buffer. Lane 4 contains refolded pkMSP-3 in standard reducing sample buffer.
(TIF)
