# Supplementary figures and images for: Expression and Evaluation of Recombinant Plasmodium knowlesi Merozoite Surface Protein-3 (MSP-3) for Detection of Human Malaria
Source: PLoS One. 2016 Jul 8;11(7):e0158998. doi: 10.1371/journal.pone.0158998 (PMC4938616; doi:10.1371/journal.pone.0158998)

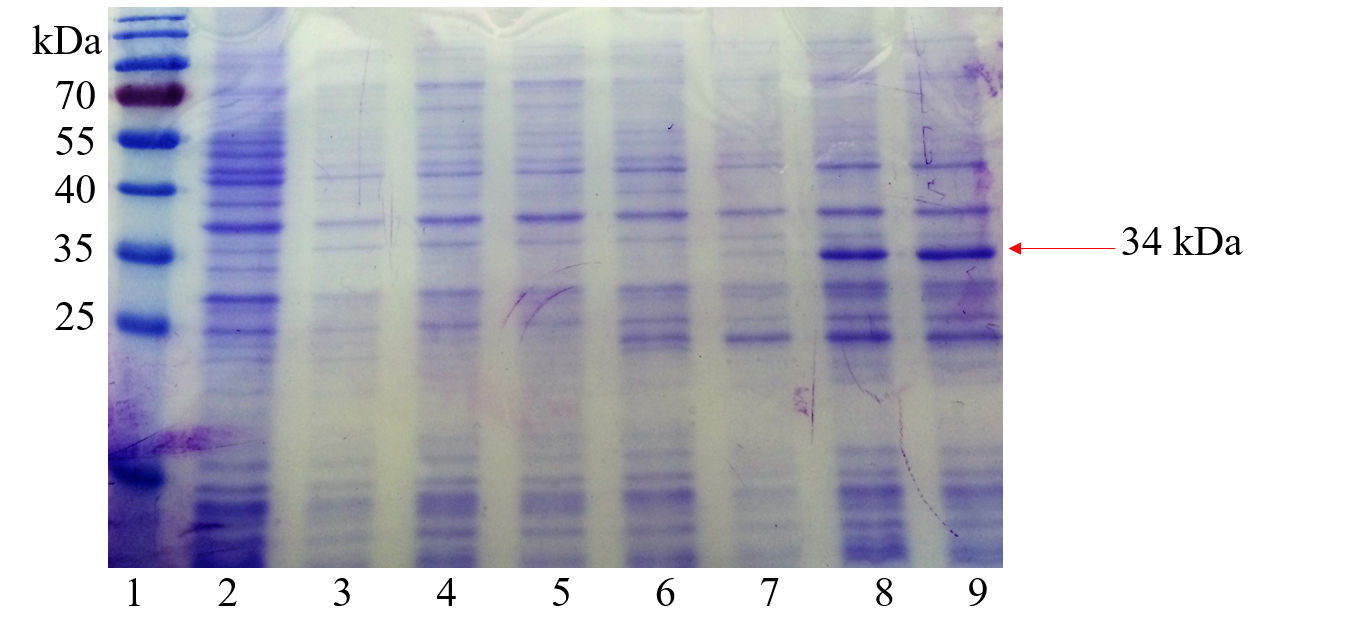

Supplement: S1 Fig — Lane 1, Bio-Rad (Hercules, CA) Pre-stained Broad Range Protein Ladder; Lane 2–5, negative control pRSET A clone induced with 0.5 mM, 0.75 mM, 1 mM and 2.0 mM IPTG; Lane 6–9, pkMSP-3 clone induced with 0.5 mM, 0.75 mM, 1 mM and 1.5 mM IPTG; The arrow indicates expression of pkMSP-3 at expected size (34 kDa) with optimum expression at 1 mM IPTG with no obvious increase in expression at higher concentrations. (TIF) [file pone.0158998.s001.tif]

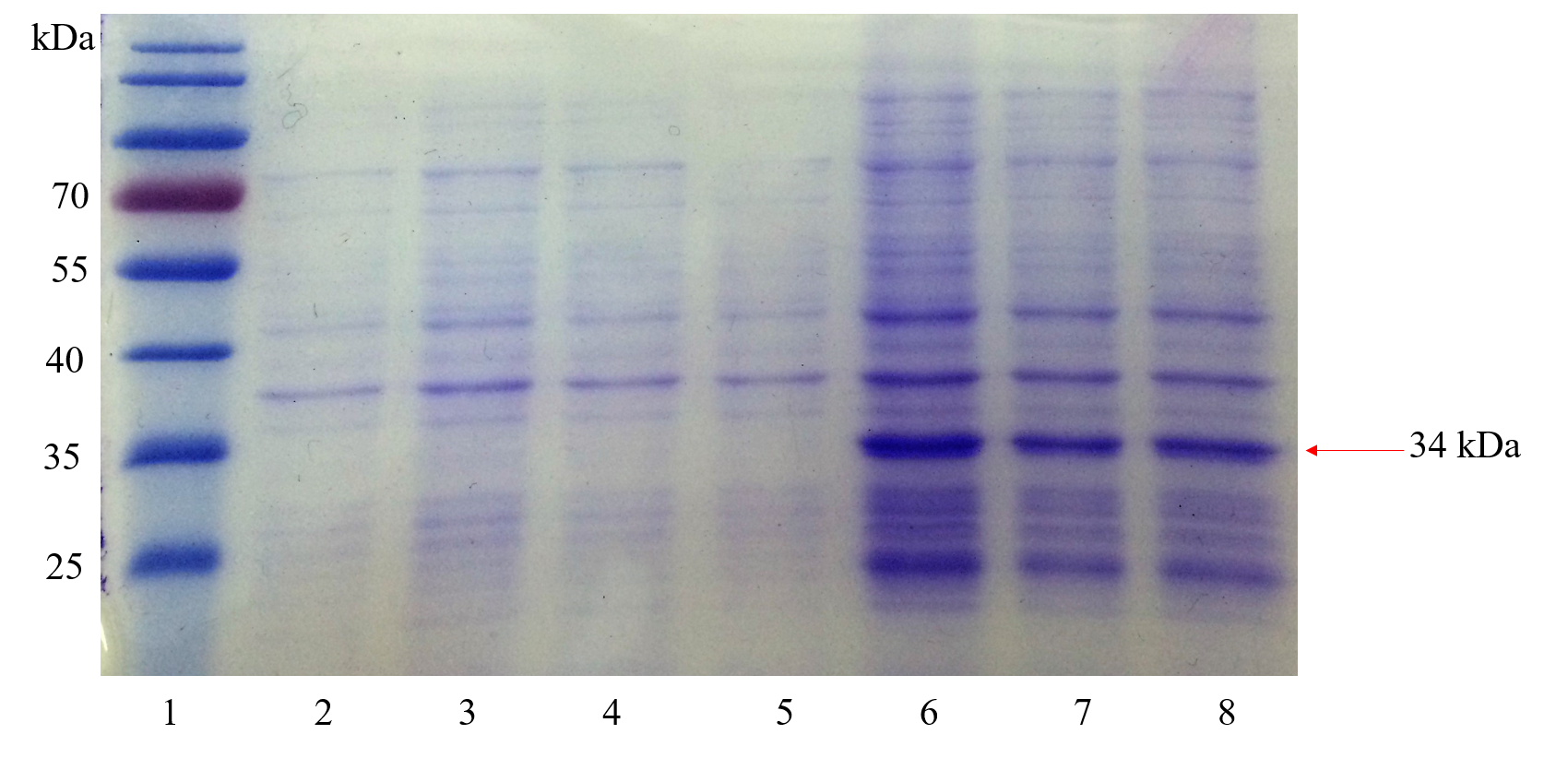

Supplement: S2 Fig — Lane 1, Bio-Rad (Hercules, CA) Pre-stained Broad Range Protein Ladder; Lane 2–5, negative control pRSET A clone induced at OD 0.4, OD 0.6, OD 0.8 and OD 1.0 with 1 mM isopropyl β-D-1-thiogalactopyranoside (IPTG); Lane 6–8, pkMSP-3 clone induced at OD 1.0, OD 0.6, and OD 0.4 with 1 mM (IPTG); The arrow indicates expression of pkMSP-3 at expected size (34 kDa) with optimum expression at 1 mM IPTG. (TIF) [file pone.0158998.s002.tif]

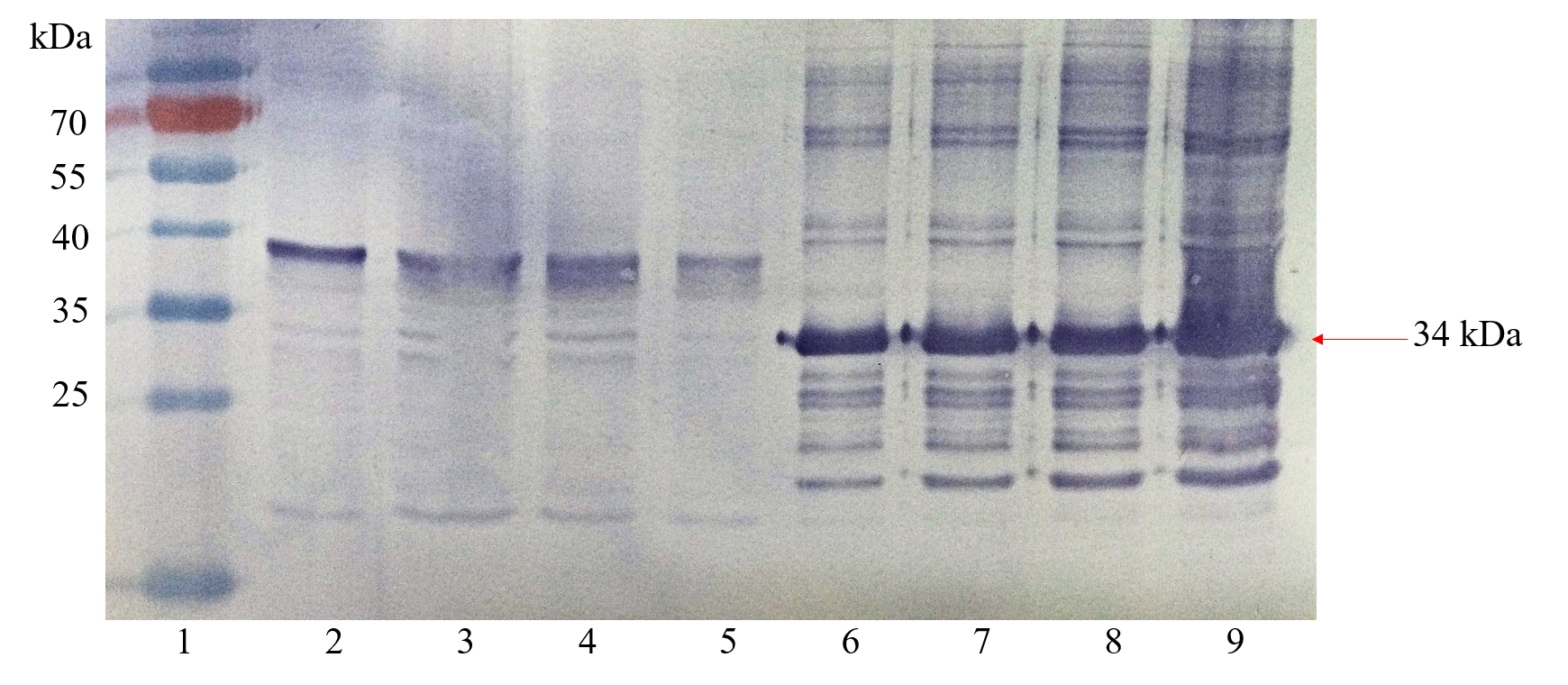

Supplement: S3 Fig — Lane 1, Bio-Rad (Hercules, CA) Pre-stained Broad Range Protein Ladder; Lane 2–5, negative control pRSET A clone induced with 0.5 mM, 0.75 mM, 1 mM and 2.0 mM IPTG; Lane 6–9, pkMSP-3 clone induced with 0.5 mM, 0.75 mM, 1 mM and 1.5 mM IPTG; The arrow indicates expression of pkMSP-3 at expected size (34 kDa). (TIF) [file pone.0158998.s003.tif]

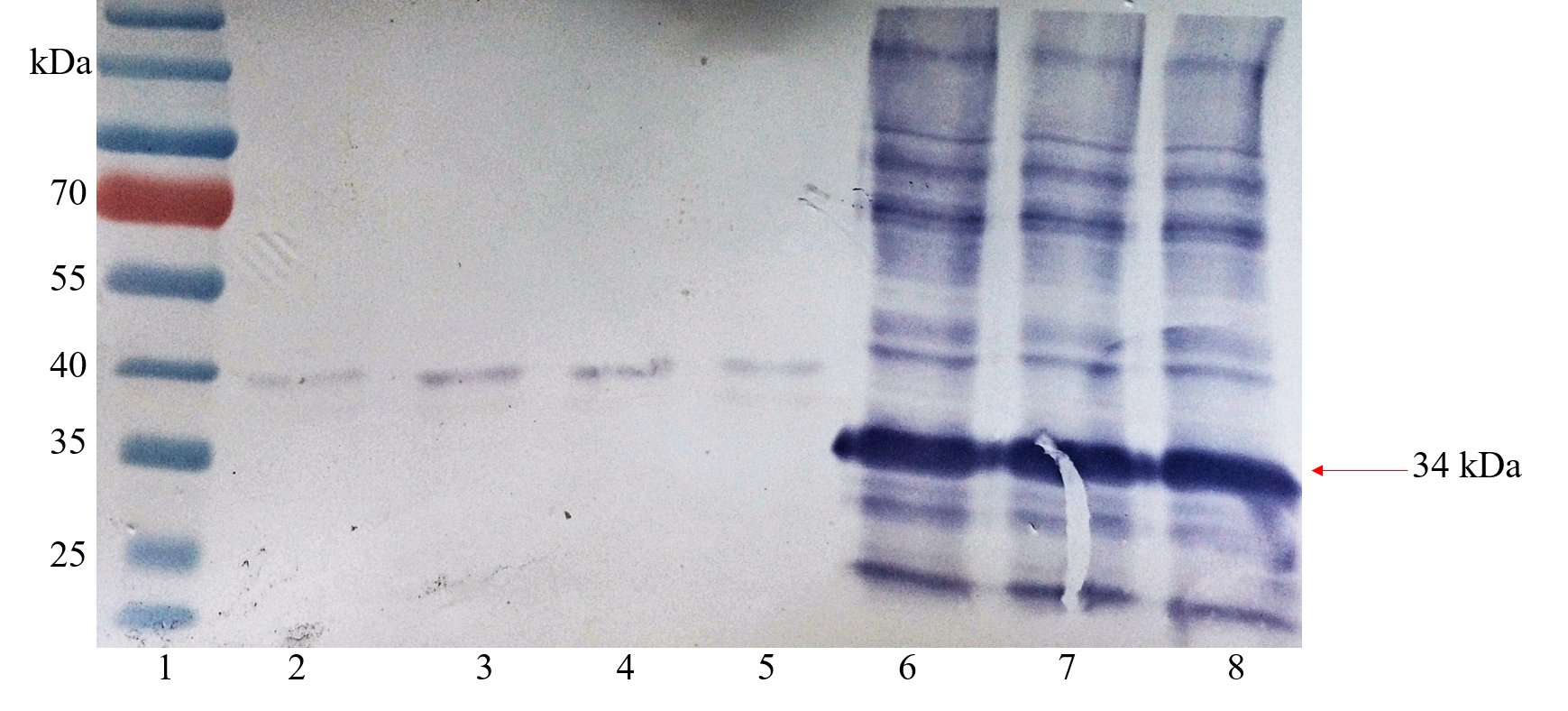

Supplement: S4 Fig — Lane 1, Bio-Rad (Hercules, CA) Pre-stained Broad Range Protein Ladder; Lane 2–5, negative control pRSET A clone induced at OD 0.4, OD 0.6, OD 0.8 and OD 1.0 with 1 mM isopropyl β-D-1-thiogalactopyranoside (IPTG); Lane 6–8, pkMSP-3 clone induced at OD 1.0, OD 0.6, and OD 0.4 with 1 mM (IPTG); The arrow indicates expression of pkMSP-3 at expected size (34 kDa). (TIF) [file pone.0158998.s004.tif]

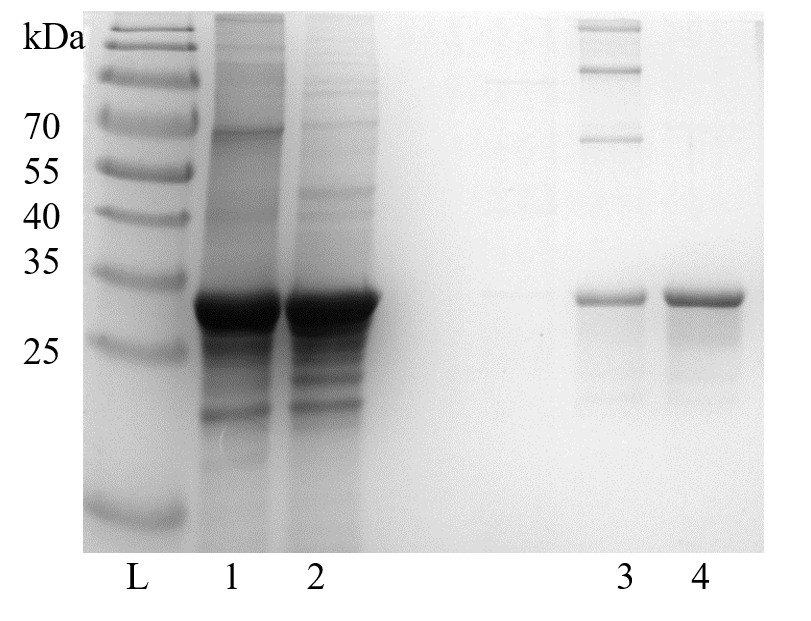

Supplement: S5 Fig — Lane 1 contains native pkMSP-3 before solubilisation and refolding in non-reducing sample buffer. Lane 2 contains native pkMSP-3 before solubilisation and refolding in standard reducing sample buffer. Lane 3 contains refolded pkMSP-3 in non-reducing sample buffer. Lane 4 contains refolded pkMSP-3 in standard reducing sample buffer. (TIF) [file pone.0158998.s005.tif]
